# Supplementary material for: Synthesis, Crystal Structures, Lipophilic Properties and Antimicrobial Activity of 5-Pyridylmethylidene-3-rhodanine-carboxyalkyl Acids Derivatives
Source: Molecules. 2022 Jun 21;27(13):3975. doi: 10.3390/molecules27133975 (PMC9268742; doi:10.3390/molecules27133975)
Supplement: Supplementary file 1 [file molecules-27-03975-s001.zip › molecules-1762532-supplementary.pdf]

molecules

Supplementary Materials

Synthesis, Crystal Structures, Lipophilic Properties and Antimicrobial Activity of 5-Pyridylmethylidene-3-Rhodanine-Carboxyalkyl Acids Derivatives

Ewa Żesławska [1], Robert Zakrzewski [2], Arkadiusz Nowicki [2], Izabela Korona-Głowniak [3], Antonín Lyčka [4], Agnieszka Kania [1], Krzysztof Kazimierz Zborowski [5], Piotr Suder [6], Agnieszka Skórska-Stania [5] and Waldemar Tejchman [1,*]

[1] Institute of Biology, Pedagogical University of Krakow, Podchorążych 2, 30-084 Kraków, Poland; ewa.zeslawska@up.krakow.pl (E.Ż.); agnieszka.kania@up.krakow.pl (A.K.)
[2] Faculty of Chemistry, University of Lodz, Tamka 12, 91-403 Łódź, Poland; robert.zakrzewski@chemia.uni.lodz.pl (R.Z.); a.nowi@o2.pl (A.N.)
[3] Department of Pharmaceutical Microbiology, Medical University of Lublin, Chodźki 1, 20-093 Lublin, Poland; iza.glowniak@umlub.pl
[4] Department of Chemistry, University of Hradec Králové, Rokitanského 62, 500 03 Hradec Králové III, Czech Republic; antonin.lycka@uhk.cz
[5] Faculty of Chemistry, Jagiellonian University in Kraków, Gronostajowa 2, 30-387 Kraków, Poland; zborowsk@chemia.uj.edu.pl (K.K.Z.); agnieszka.skorska-stania@uj.edu.pl (A.S.-S.)
[6] Department of Analytical Chemistry and Biochemistry, Faculty of Materials Science and Ceramics, AGH University of Science and Technology, Mickiewicza 30, 30-059 Kraków, Poland; piotr.suder@agh.edu.pl

Table S1. Relationship between the retention factor (R_M) and organic modifier content in the mobile phase obtained using RP-TLC.
Table S2. Calculated LogP values from online databases.
Table S3. Physicochemical properties of the tested compounds obtained *in-silico*.
Table S4. Bioactivity parameters of the tested compounds obtained from the Molinspiration Internet database.
Table S5. Antimicrobial activity of compounds /**3a – 3d**/.
Table S6. Antimicrobial activity of compounds /**4a – 4d**/.
Table S7. Antimicrobial activity of compounds /**5a – 5d**/.
Table S8. ^{1}H, ^{13}C and ^{15}N chemical shifts in compounds /**3a,b,c,d – 5a,b,c,d**/ in DMSO-D_6.
Table S9. Crystal data and structure refinement for /**3c**/.
Table S10. Crystal data and structure refinement for /**4a**/.
Table S11. Crystal data and structure refinement for /**5a**/.

1. Correlation of R_M^0 and miLogP

Figure S1. Relationship between miLogP (Molinspiration) and R_M^0 (RP-TLC) values.

2. Other correlations between experimental and *in-silico* data

Table S12. Regression coefficients and Pearson correlation coefficient for the relation between the values obtained *in-silico* and experimental value R_M^0.
Table S13. Regression coefficients and Pearson correlation coefficient for the relation between the values obtained *in-silico* and experimental value *A* (slope of the straight).

3. Correlations between in-silico results

Table S14. Values of R^2 obtained by comparing in-silico results.

Table S1. Relationship between the retention factor (R_M) and organic modifier content in the mobile phase obtained using RP-TLC.

Organic modifier	Content of organic modifier in the mobile phase, %	Compound	R_M^0	SD R_M^0	A	SD A	R^2
acetonitrile	40-100	**3a**	1.7999	0.2006	-0.0251	0.00276	0.9432
	-	**4a**	-	-	-	-	-
	30-90	**5a**	1.9925	0.2527	-0.0283	0.00400	0.9092
	40-100	**3b**	2.0517	0.1867	-0.0267	0.00256	0.9560
	30-90	**4b**	2.0458	0.2300	-0.0290	0.00364	0.9273
	30-90	**5b**	2.2748	0.2403	-0.0301	0.00380	0.9261
	40-100	**3c**	2.3689	0.2218	-0.0290	0.00305	0.9478
	40-100	**4c**	1.3658	0.4467	-0.0156	0.00614	0.5626
	40-90	**5c**	2.3507	0.3138	-0.0292	0.00467	0.9073
	60-100	**3d**	3.2552	0.2318	-0.0324	0.00285	0.9773
	50-100	**4d**	2.4344	0.3686	-0.0207	0.00479	0.8234
	50-90	**5d**	3.4900	0.2878	-0.0338	0.00403	0.9591
methanol	50-100	**3a**	4.0038	0.2835	-0.0453	0.00369	0.9742
	-	**4a**	-	-	-	-	-
	30-100	**5a**	2.5845	0.0890	-0.0307	0.00129	0.9895
	60-100	**3b**	4.0195	0.2760	-0.0444	0.00340	0.9827
	50-100	**4b**	3.3383	0.2097	-0.0377	0.00273	0.9796
	40-100	**5b**	2.9838	0.0874	-0.0341	0.00120	0.9938
	60-100	**3c**	5.1059	0.4312	-0.0555	0.00531	0.9733
	60-100	**4c**	3.8318	0.2361	-0.0419	0.00291	0.9858
	50-100	**5c**	3.6553	0.1499	-0.0407	0.00195	0.9909
	70-100	**3d**	6.2760	0.2458	-0.0626	0.00287	0.9958
	70-100	**4d**	6.4025	0.3572	-0.0637	0.00417	0.9915
	80-100	**5d**	5.4235	0.4998	-0.0546	0.00553	0.9898
acetone	40-100	**3a**	3.0990	0.2174	-0.0405	0.00299	0.9736
	30-90	**4a**	2.5086	0.0627	-0.0372	0.00099	0.9965
	30-100	**5a**	2.0087	0.3356	-0.0278	0.00487	0.8444
	50-100	**3b**	3.0261	0.2339	-0.0394	0.00304	0.9767
	30-100	**4b**	2.5265	0.2220	-0.0347	0.00322	0.9509
	40-100	**5b**	2.3070	0.4566	-0.0304	0.00627	0.8240
	50-100	**3c**	3.3633	0.2100	-0.0423	0.00273	0.9836
	40-100	**4c**	2.8126	0.3352	-0.0371	0.00460	0.9284
	40-100	**5c**	2.6197	0.4544	-0.0334	0.00624	0.8513
	60-100	**3d**	4.8853	0.3986	-0.0559	0.00491	0.9774
	60-100	**4d**	4.0406	0.5622	-0.0469	0.00692	0.9387
	60-100	**5d**	3.3140	0.6482	-0.0369	0.00798	0.8772
propan-2-ol	30-100	**3a**	1.6422	0.2371	-0.0250	0.00344	0.8978
	-	**4a**	-	-	-	-	-
	30-100	**5a**	1.2167	0.2453	-0.0199	0.00356	0.8391
	30-100	**3b**	1.8585	0.2632	-0.0272	0.00382	0.8939
	30-100	**4b**	1.4525	0.2800	-0.0217	0.00406	0.8266
	30-100	**5b**	1.4285	0.2525	-0.0218	0.00366	0.8552
	30-100	**3c**	2.1034	0.2815	-0.0295	0.00408	0.8966
	30-100	**4c**	1.7383	0.3068	-0.0247	0.00445	0.8368
	30-100	**5c**	1.8758	0.3327	-0.0267	0.00483	0.8355
	40-100	**3d**	3.1220	0.3342	-0.0375	0.00459	0.9304
	40-100	**4d**	2.5721	0.2999	-0.0304	0.00412	0.9161
	40-100	**5d**	2.5498	0.2467	-0.0303	0.00339	0.9412
1,4-dioxane	40-100	**3a**	3.7148	0.2372	-0.0555	0.00385	0.9858
	30-100	**4a**	2.7832	0.0634	-0.0438	0.00110	0.9975

30-100	**5a**	2.9443	0.1315	-0.0470	0.00191	0.9902
50-100	**3b**	3.6615	0.2640	-0.0538	0.00400	0.9890
30-100	**4b**	3.2709	0.0722	-0.0494	0.00105	0.9973
30-100	**5b**	3.3744	0.1432	-0.0514	0.00208	0.9903
50-100	**3c**	3.9814	0.2816	-0.0570	0.00427	0.9889
40-100	**4c**	3.5351	0.1194	-0.0518	0.00164	0.9950
40-100	**5c**	3.7556	0.1855	-0.0555	0.00255	0.9896
60-100	**3d**	5.5999	0.5303	-0.0727	0.00752	0.9894
60-100	**4d**	5.3824	0.3383	-0.0707	0.00416	0.9897
60-100	**5d**	5.2828	0.3551	-0.0705	0.00437	0.9886

Table S2. Calculated LogP values from online databases.

**Compound**	**Programme**				
	**ALOGPs**	**AClogP**	**XlogP2**	**XLOGP3**	**$LogP_{ACD}$**
**3a**	2.08	1.49	0.56	0.56	0.53
**4a**	1.82	1.39	0.47	0.47	0.65
**5a**	1.84	1.39	0.47	0.47	0.40
**3b**	2.39	1.96	0.92	2.67	0.83
**4b**	2.16	1.85	0.83	2.63	0.95
**5b**	2.19	1.85	0.83	2.63	0.70
**3c**	2.78	2.42	1.28	3.03	1.06
**4c**	2.46	2.32	1.19	2.99	1.18
**5c**	2.47	2.32	1.19	2.99	0.93
**3d**	4.77	4.74	4.12	5.73	3.72
**4d**	4.54	4.64	4.03	5.70	3.84
**5d**	4.57	4.64	4.03	5.70	3.59

Table S3. Physicochemical properties of the tested compounds obtained *in-silico*.

**Compound**	**Physicochemical properties**							
	**miLogP**	**TPSA**	**MW**	**NOHBA**	**NOHBD**	**V**	**NORB**	**NV**
**3a**	0.51	72.20	308.38	5	1	251.63	5	0
**4a**	0.44	72.20	308.38	5	1	251.63	5	0
**5a**	0.39	72.20	308.38	5	1	251.63	5	0
**3b**	1.01	72.20	322.41	5	1	268.43	6	0
**4b**	0.95	72.20	322.41	5	1	268.43	6	0
**5b**	0.90	72.20	322.41	5	1	268.43	6	0
**3c**	1.52	72.20	336.44	5	1	285.23	7	0
**4c**	1.45	72.20	336.44	5	1	285.23	7	0
**5c**	1.40	72.20	336.44	5	1	285.23	7	0
**3d**	4.05	72.20	406.57	5	1	369.24	12	0
**4d**	3.98	72.20	406.57	5	1	369.24	12	0
**5d**	3.93	72.20	406.57	5	1	369.24	12	0

Table S4. Bioactivity parameters of the tested compounds obtained from the Molinspiration Internet database.

Compound	Bioactivity parameters					
	GPCR ligand	Ion channel modulator	Kinase inhibitor	Nuclear receptor ligand	Protease inhibitor	Enzyme inhibitor
**3a**	-0.75	-1.60	-0.87	-0.70	-0.56	-0.12
**4a**	-0.68	-1.60	-0.74	-0.78	-0.55	-0.12
**5a**	-0.71	-1.61	-0.73	-0.69	-0.48	-0.10
**3b**	-0.66	-1.52	-0.79	-0.62	-0.47	-0.10
**4b**	-0.60	-1.52	-0.64	-0.69	-0.46	-0.09
**5b**	-0.62	-1.52	-0.67	-0.61	-0.40	-0.07
**3c**	-0.60	-1.45	-0.73	-0.56	-0.41	-0.09
**4c**	-0.55	-1.45	-0.59	-0.63	-0.40	-0.09
**5c**	-0.57	-1.46	-0.61	-0.55	-0.34	-0.07
**3d**	-0.47	-1.18	-0.58	-0.43	-0.29	-0.08
**4d**	-0.43	-1.18	-0.46	-0.49	-0.28	-0.07
**5d**	-0.45	-1.19	-0.48	-0.43	-0.23	-0.06

Table S5. Antimicrobial activity of compounds /**3a – 3d**/.

Chemicals / Microorganism	3a		3b		3c		3d	
	MIC (µg/ml)	MBC (µg/ml)	MIC (µg/ml)	MBC (µg/ml)	MIC (µg/ml)	MBC (µg/ml)	MIC (µg/ml)	MBC (µg/ml)
**Gram-positive bacteria**								
S. aureus ATCC 25923	**62.5**	>1000	**62.5**	>1000	**62.5**	>1000	**62.5**	>1000
S. aureus ATCC 6538	**125**	>1000	**62.5**	>1000	**62.5**	>1000	**62.5**	>1000
S. aureus ATCC 43300	**62.5**	>1000	**125**	>1000	**62.5**	>1000	**62.5**	>1000
S. epidermidis ATCC 12228	**125**	>1000	**62.5**	>1000	**62.5**	>1000	**31.3**	>1000
M. luteus ATCC 10240	**125**	>1000	**62.5**	>1000	**125**	>1000	<u>7.8</u>	<u>7.8</u>
B. subtilis ATCC 6633	**62.5**	>1000	**62.5**	>1000	**31.3**	>1000	**125**	>1000
B. cereus ATCC 10876	**125**	>1000	**62.5**	>1000	**31.3**	>1000	**62.5**	>1000
S. pyogenes ATCC 19615	500	>1000	>1000	>1000	>1000	>1000	1000	>1000
S. pneumoniae ATCC 49619	1000	>1000	>1000	>1000	>1000	>1000	>1000	>1000
S. mutans ATCC 25175	500	>1000	>1000	>1000	>1000	>1000	>1000	>1000
**Gram-negative bacteria**								
S. typhimurium ATCC 14028	>1000	>1000	>1000	>1000	>1000	>1000	>1000	>1000
E. coli ATCC 25922	>1000	>1000	>1000	>1000	>1000	>1000	>1000	>1000
P. mirabilis ATCC 12453	250	>1000	>1000	>1000	>1000	>1000	250	>1000
K. pneumoniae ATCC 13883	250	>1000	>1000	>1000	>1000	>1000	>1000	>1000
P. aeruginosa ATCC 9027	>1000	>1000	>1000	>1000	>1000	>1000	125	>1000
**Yeasts**								
C. albicans ATCC 102231	>1000	>1000	>1000	>1000	500	>1000	>1000	>1000
C. albicans ATCC 2091	>1000	>1000	1000	>1000	125	>1000	>1000	>1000
C. parapsilosis ATCC 22019	>1000	>1000	>1000	>1000	125	>1000	>1000	>1000
C. glabrata ATCC 90030	>1000	>1000	>1000	>1000	500	>1000	>1000	>1000
C. krusei ATCC 14243	>1000	>1000	500	>1000	500	>1000	>1000	>1000

Table S6. Antimicrobial activity of compounds **/4a – 4d/**.

Chemicals / Microorganism	4a		4b		4c		4d	
	MIC (µg/ml)	MBC (µg/ml)	MIC (µg/ml)	MBC (µg/ml)	MIC (µg/ml)	MBC (µg/ml)	MIC (µg/ml)	MBC (µg/ml)
**Gram-positive bacteria**								
S. aureus ATCC 25923	**250**	>1000	**250**	>1000	**250**	>1000	**500**	>1000
S. aureus ATCC 6538	**250**	>1000	**250**	>1000	**250**	>1000	**500**	>1000
S. aureus ATCC 43300	**250**	>1000	**250**	>1000	**250**	>1000	**250**	>1000
S. epidermidis ATCC 12228	**250**	>1000	**125**	>1000	**125**	>1000	**250**	>1000
M. luteus ATCC 10240	**250**	>1000	**15.6**	>1000	**31.3**	>1000	**31.3**	>1000
B. subtilis ATCC 6633	**250**	>1000	**250**	500	**125**	>1000	**500**	31.3
B. cereus ATCC 10876	**250**	>1000	**125**	>1000	**125**	>1000	**62.5**	>1000
S. pyogenes ATCC 19615	**>1000**	>1000	**500**	>1000	**>1000**	>1000	**>1000**	>1000
S. pneumoniae ATCC 49619	>1000	>1000	500	>1000	>1000	>1000	>1000	>1000
S. mutans ATCC 25175	>1000	>1000	>1000	>1000	>1000	>1000	>1000	>1000
**Gram-negative bacteria**								
S. typhimurium ATCC 14028	>1000	>1000	>1000	>1000	>1000	>1000	>1000	>1000
E. coli ATCC 25922	>1000	>1000	>1000	>1000	>1000	>1000	>1000	>1000
P. mirabilis ATCC 12453	>1000	>1000	>1000	>1000	>1000	>1000	>1000	>1000
K. pneumoniae ATCC 13883	>1000	>1000	>1000	>1000	>1000	>1000	>1000	>1000
P. aeruginosa ATCC 9027	>1000	>1000	>1000	>1000	>1000	1000	>1000	>1000
**Yeasts**								
C. albicans ATCC 102231	>1000	>1000	1000	>1000	>1000	>1000	1000	>1000
C. albicans ATCC 2091	>1000	>1000	>1000	>1000	>1000	>1000	500	>1000
C. parapsilosis ATCC 22019	>1000	>1000	>1000	>1000	>1000	>1000	500	>1000
C. glabrata ATCC 90030	>1000	>1000	>1000	>1000	>1000	>1000	1000	>1000
C. krusei ATCC 14243	>1000	>1000	1000	>1000	>1000	>1000	1000	>1000

Table S7. Antimicrobial activity of compounds **/5a – 5d/**.

Chemicals / Microorganism	5a		5b		5c		5d	
	MIC (µg/ml)	MBC (µg/ml)	MIC (µg/ml)	MBC (µg/ml)	MIC (µg/ml)	MBC (µg/ml)	MIC (µg/ml)	MBC (µg/ml)
**Gram-positive bacteria**								
S. aureus ATCC 25923	**500**	>1000	**250**	>1000	**125**	>1000	**250**	>1000
S. aureus ATCC 6538	**500**	>1000	**250**	>1000	**125**	>1000	**250**	>1000
S. aureus ATCC 43300	**500**	>1000	**250**	>1000	**125**	>1000	**250**	>1000
S. epidermidis ATCC 12228	**125**	>1000	**125**	>1000	**125**	>1000	**125**	>1000
M. luteus ATCC 10240	**125**	>1000	**7.8**	>1000	**31.3**	>1000	**7.8**	>1000
B. subtilis ATCC 6633	**500**	500	**>1000**	>1000	**125**	>1000	**500**	>1000
B. cereus ATCC 10876	**250**	>1000	**>1000**	>1000	**62.5**	>1000	**125**	>1000
S. pyogenes ATCC 19615	500	>1000	500	>1000	250	>1000	1000	>1000
S. pneumoniae ATCC 49619	1000	>1000	>1000	>1000	1000	>1000	>1000	>1000
S. mutans ATCC 25175	1000	>1000	>1000	>1000	>1000	>1000	>1000	>1000
**Gram-negative bacteria**								

S. typhimurium ATCC 14028	>1000	>1000	>1000	>1000	>1000	>1000	>1000	>1000
E. coli ATCC 25922	>1000	>1000	>1000	>1000	>1000	>1000	>1000	>1000
P. mirabilis ATCC 12453	>1000	>1000	>1000	>1000	>1000	>1000	>1000	>1000
K. pneumoniae ATCC 13883	>1000	>1000	>1000	>1000	>1000	>1000	>1000	>1000
P. aeruginosa ATCC 9027	>1000	>1000	>1000	>1000	>1000	>1000	125	>1000
**Yeasts**								
C. albicans ATCC 102231	500	>1000	1000	>1000	500	>1000	>1000	>1000
C. albicans ATCC 2091	500	>1000	250	>1000	1000	>1000	>1000	>1000
C. parapsilosis ATCC 22019	1000	>1000	>1000	>1000	1000	>1000	>1000	>1000
C. glabrata ATCC 90030	500	>1000	>1000	>1000	>1000	>1000	1000	>1000
C. krusei ATCC 14243	>1000	>1000	>1000	>1000	>1000	>1000	1000	>1000

Table S8. ^{1}H, ^{13}C and ^{15}N chemical shifts in compounds **/3a,b,c,d – 5a,b,c,d/** in DMSO-D_6.

	**3a**	**4a**	**5a**	**3b**	**4b**	**5b**	**3c**	**4c**	**5c**	**3d**	**4d**	**5d**
C=S	-/200.1	-/193.4	-/193.4	-/199.9	-/193.2	-/193.2	-/199.8	-/193.2	-/193.1	-/199.8	-/192.9	-/193.1
N	-/-186.7 [a]	-/-186.1 [a]	-/-186.6 [a]	-/-185.8 [a]	-/-185.9 [a]	-/-185.8 [a]	-/-185.4 [a]	-/-185.3 [a]	-/-185.3 [a]	-/-185.3 [a]	-/-185.3 [a]	-/-185.6 [a]
NCH_2	4.07/43.2	4.08/43.8	4.07/43.9	4.03/43.4	4.04/44.1	4.03/44.1	4.01/43.6	4.02/44.2	4.01/44.3	4.00/43.7	3.98/44.3	4.01/44.4
CH_2	1.88/22.1	1.91/22.0	1.88/22.0	1.65/21.8	1.67/21.7	1.66/21.7	1.64/26.2	1.64/26.1	1.64/26.1	1.61/26.4	1.60/26.3	1.63/26.2
CH_2	-	-	-	-	-	-	1.29/25.7	1.29/25.7	1.29/25.7	b	c	d
CH_2	-	-	-	1.51/26.1	1.52/26.0	1.51/26.0	1.52/24.1	1.52/24.1	1.53/24.0	1.45/24.5	1.45/24.5	1.47/24.5
CH_2	2.29/31.0	2.30/31.0	2.31/31.0	2.25/33.1	2.24/33.1	2.22/33.1	2.20/33.4	2.20/33.4	2.20/33.4	2.16/33.7	2.15/33.7	2.17/33.7
COOH	12.14/173.8	12.13/173.8	12.15/173.8	12.07/174.2	12.07/174.2	12.07/174.2	12.02/174.4	12.05/174.4	12.02/174.4	11.79/174.5	11.98/174.5	11.97/174.5
C=O	-/167.2	-/167.0	-/167.0	-/167.1	-/168.6	-/166.8	-/167.1	-/166.8	-/166.8	-/167.1	-/166.7	-/166.8
C=	-/126.9	-/124.8	-/127.7	-/126.7	-/124.7	-/127.6	-/126.7	-/124.7	-/127.5	-/128.5	-/124.6	-/127.5
=CH-	7.82/128.4	7.84/129.2	7.75/129.2	7.84/128.6	7.86/129.5	7.78/129.5	7.85/128.6	7.87/129.5	7.78/129.5	7.82/128.5	7.81/129.4	7.78/129.5
N	-/-68.9 [a]	-/-63.5 [a]	-/-59.8 [a]	-/-68.8 [a]	-/-63.2 [a]	-/-58 0.6 [a]	-/-68.8 [a]	-/-63.2 [a]	-/-59.1 [a]	-/-68.6 [a]	-/-63.5 [a]	-/-58.8 [a]
2	-/151.2	8.88/151.9	8.73/150.8	-/151.2	8.88/151.9	8.74/150.8	-/151.2	8.89/151.9	8.74/150.8	-/151.2	8.84/151.9	8.74/150.8
3	7.93/128.3	-/129.2	7.57/123.7	7.93/128.3	-/129.2	7.57/123.7	7.94/128.3	-/129.2	7.57/123.7	7.93/128.3	-/129.1	7.57/123.7
4	7.96/137.7	7.98/136.5	-/140.0	7.97/137.7	7.99/136.5	-/139.9	7.97/137.7	7.99/0136.5	-/139.9	7.96/137.7	7.94/136.5	-/139.9
5	7.45/124.2	7.58/124.4	7.57/123.7	7.46/124.2	7.58/124.4	7.57/123.7	7.46/124.2	7.59/124.4	7.57/123.7	7.44/124.2	7.55/124.3	7.57/123.7
6	8.80/149.6	8.62/150.9	8.73/150.8	8.81/149.6	8.64/150.9	8.74/150.8	8.81/149.6	8.66/151.0	8.74/150.8	8.79/149.5	8.63/150.9	8.74/150.8

[a] ◎(^{15}N)

[b] ◎(^{1}H) = 1.13-1.28 (12H, m), (^{13}C) = 28.85, 28.81, 28.76, 28.57, 28.55, 26.17

[c] ◎(^{1}H) = 1.14-1.28 (12H, m), (^{13}C) = 28.90, 28.86, 28.81, 28.61, 28.59, 26.19

[d] ◎(^{1}H) = 1.14-1.28 (12H, m), (^{13}C) = 28.84, 28.78, 28.75, 28.55, 28.53, 26.24

Table S9. Crystal data and structure refinement for /**3c**/. .

**Empirical formula**	$\mathbf{C_{15}H_{16}N_2O_3S_2}$	
Formula weight	336.42	
Temperature	130(2) K	
Wavelength	0.71073 Å	
Crystal system	Triclinic	
Space group	P -1	
Unit cell dimensions	a = 5.0766(3) Å	α = 98.784(5)°.
	b = 12.3912(8) Å	β = 93.967(5)°.
	c = 12.6046(8) Å	γ = 90.083(5)°.
Volume	781.66(9) $Å^3$	
Z	2	
Density (calculated)	1.429 Mg/m^3	
Absorption coefficient	0.354 mm-1	
F(000)	352	
Crystal size	0.520 x 0.200 x 0.030 mm^3	
Theta range for data collection	2.508 to 27.987°.	
Index ranges	-6<=h<=6, -15<=k<=16, -16<=l<=16	
Reflections collected	10738	
Independent reflections	3716 [R(int) = 0.0386]	
Completeness to theta = 25.242°	99.8 %	
Refinement method	Full-matrix least-squares on F^2	
Data / restraints / parameters	3716 / 0 / 203	
Goodness-of-fit on F^2	1.138	
Final R indices [I>2sigma(I)]	R1 = 0.0497, wR2 = 0.1171	
R indices (all data)	R1 = 0.0706, wR2 = 0.1274	
Extinction coefficient	n/a	
Largest diff. peak and hole	0.457 and -0.299 e.$Å^{-3}$	

Table S10. Crystal data and structure refinement for /**4a**/.

**Empirical formula**	$\mathbf{C_{13}H_{12}N_2O_3S_2}$	
Formula weight	308.37	
Temperature	130(2) K	
Wavelength	0.71073 Å	
Crystal system	Triclinic	
Space group	P -1	
Unit cell dimensions	a = 5.3749(3) Å	α = 73.323(6)°
	b = 9.8192(6) Å	β = 78.924(5)°
	c = 13.1564(9) Å	γ = 82.765(7)°
Volume	650.91(7) $Å^3$	
Z	2	
Density (calculated)	1.573 Mg/m^3	
Absorption coefficient	0.417 mm^{-1}	
F(000)	320	
Crystal size	0.310 x 0.110 x 0.030 mm^3	
Theta range for data collection	3.053 to 28.691°.	
Index ranges	-7<=h<=7, -13<=k<=12, -17<=l<=16	
Reflections collected	8719	
Independent reflections	3050 [R(int) = 0.0372]	
Completeness to theta = 25.242°	99.8 %	
Refinement method	Full-matrix least-squares on F^2	
Data / restraints / parameters	3050 / 0 / 185	
Goodness-of-fit on F^2	1.071	

Final R indices [I>2sigma(I)]	R1 = 0.0408, wR2 = 0.1016	
R indices (all data)	R1 = 0.0536, wR2 = 0.1090	
Extinction coefficient	n/a	
Largest diff. peak and hole	0.483 and -0.330 e.Å^{-3}	

Table S11. Crystal data and structure refinement for /**5a**/.

**Empirical formula**	**$C_{13}H_{12}N_2O_3S_2$**	
Formula weight	308.37	
Temperature	293(2) K	
Wavelength	0.71073 Å	
Crystal system	Monoclinic	
Space group	P 21/c	
Unit cell dimensions	a = 11.5626(2) Å	α = 90°
	b = 7.9659(2) Å	β = 90.169(2)°
	c = 14.5520(3) Å	γ = 90°
Volume	1340.33(5) Å^3	
Z	4	
Density (calculated)	1.528 Mg/m^3	
Absorption coefficient	0.405 mm^{-1}	
F(000)	640	
Crystal size	0.31 x 0.25 x 0.05 mm^3	
Theta range for data collection	2.799 to 28.693°.	
Index ranges	-15<=h<=15, -10<=k<=10, -19<=l<=19	
Reflections collected	56894	
Independent reflections	3370 [R(int) = 0.0459]	
Completeness to theta = 25.242°	99.9 %	
Refinement method	Full-matrix least-squares on F^2	
Data / restraints / parameters	3370 / 0 / 185	
Goodness-of-fit on F^2	1.039	
Final R indices [I>2sigma(I)]	R1 = 0.0297, wR2 = 0.0707	
R indices (all data)	R1 = 0.0376, wR2 = 0.0762	
Extinction coefficient	n/a	
Largest diff. peak and hole	0.331 and -0.227 e.Å^{-3}	

1. Correlation of R_M^0 and miLogP

Twelve miLogP values calculated using Molinspiration internet database were correlated with R_M^0 values obtained experimentally. Correlation charts for all tested solvents are shown in Figure S1.

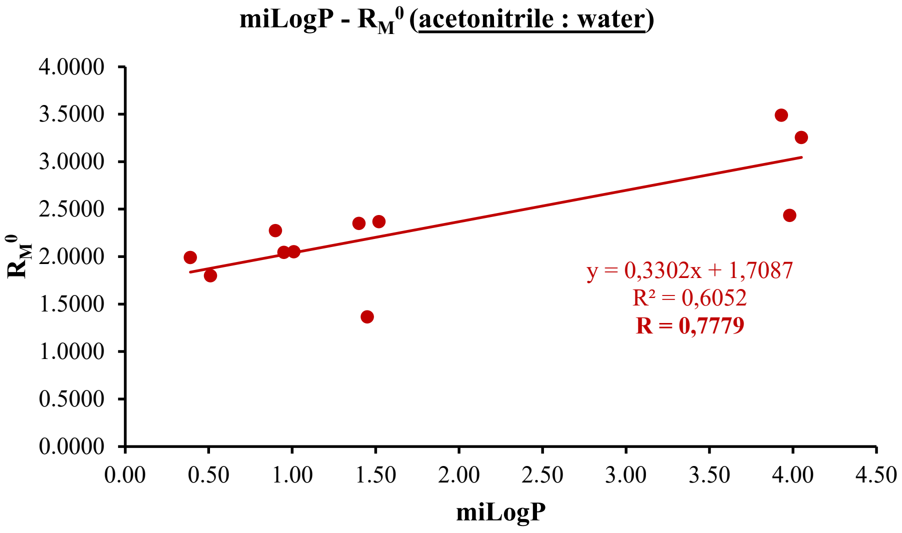
miLogP - RM0 (acetonitrile : water)
RM0
miLogP
4.0000
3.5000
3.0000
2.5000
2.0000
1.5000
1.0000
0.5000
0.0000
0.00
0.50
1.00
1.50
2.00
2.50
3.00
3.50
4.00
4.50
y = 0,3302x + 1,7087
R² = 0,6052
R = 0,7779

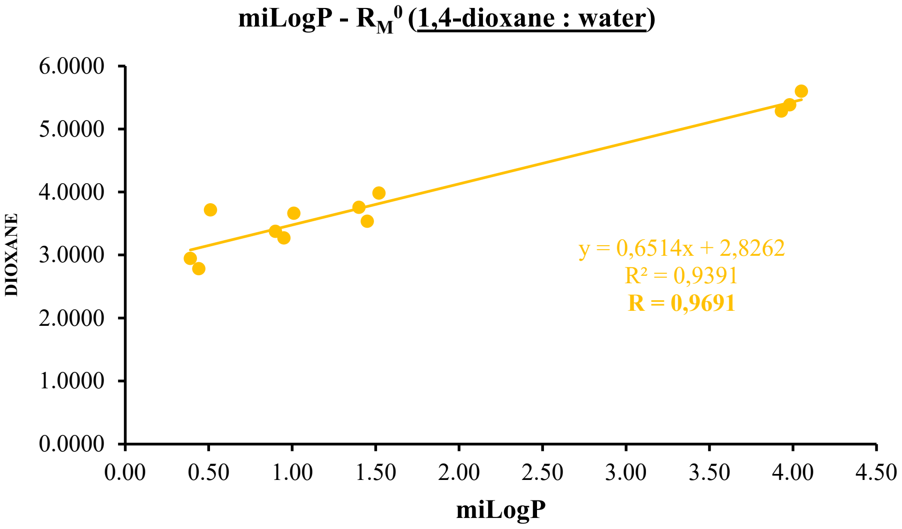
miLogP - RM0 (1,4-dioxane : water)
DIOXANE
miLogP
6.0000
5.0000
4.0000
3.0000
2.0000
1.0000
0.0000
0.00
0.50
1.00
1.50
2.00
2.50
3.00
3.50
4.00
4.50
y = 0,6514x + 2,8262
R² = 0,9391
R = 0,9691

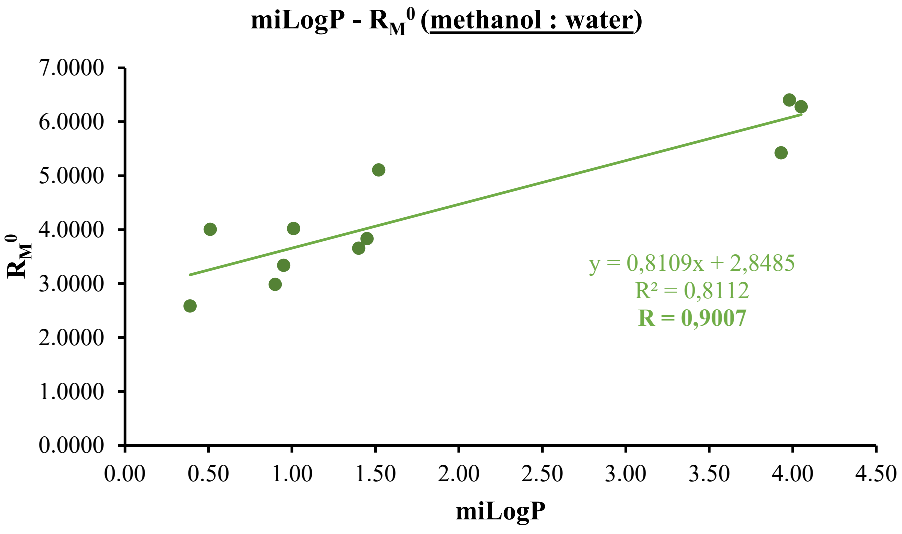
miLogP - RM0 (methanol : water)
RM0
miLogP
7.0000
6.0000
5.0000
4.0000
3.0000
2.0000
1.0000
0.0000
0.00
0.50
1.00
1.50
2.00
2.50
3.00
3.50
4.00
4.50
y = 0,8109x + 2,8485
R² = 0,8112
R = 0,9007

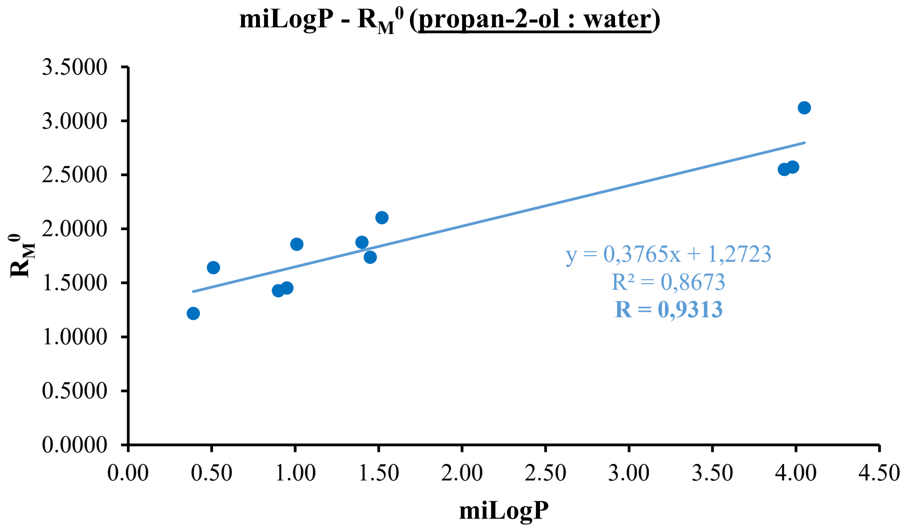

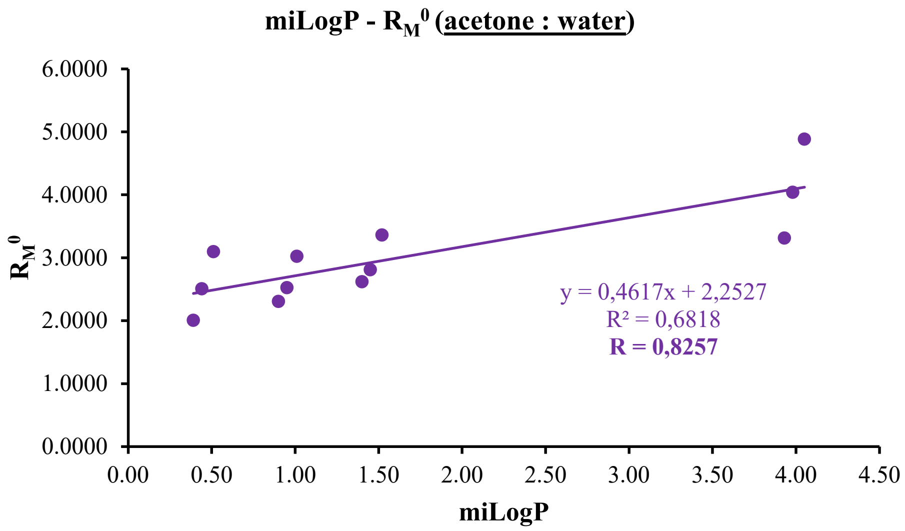

Figure S1. Relationship between miLogP (Molinspiration) and R_M^0 (RP-TLC) values.

All organic modifiers of the mobile phase show significantly statistical relationships and correlate with each other. The highest value of Pearson R coefficient was observed for the organic modifier 1,4-dioxane and the lowest one for acetonitrile and it is 0.9691 and 0.7779, respectively. A very high positive correlation is visible for modifiers such as 1,4-dioxane, methanol and propan-2-ol (Pearson's R-factor > 0.9000) and a high correlation for modifiers such as acetonitrile and acetone (0.7000 < R < 0.9000). [1]

It can be observed that all compounds in each diagram are arranged into four groups depending on the length of alkyl chain in the molecular structure.

2. Other correlations between experimental and *in-silico* data

Calculated theoretically five LogP values [2-6] were correlated with experimental values such as R_M^0 and the slope coefficient of straight a (equation 3). The results obtained (regression coefficients a, b, R^2 and Pearson's coefficient R) are shown in Table S12 and Table S13.

Table S12. Regression coefficients and Pearson correlation coefficient for the relation between the values obtained *in-silico* and experimental value R_M^0.

R_M^0 = a · LogP + b						
**Programme**	**Regression coefficients**	**acetonitrile**	**methanol**	**acetone**	**propan-2-ol**	**1,4-dioxane**
ALOGPs	*a*	0.4364	1.0627	0.6118	0.4919	0.8452
	b	1.0324	1.2140	1.3056	0.5178	1.5409
	R^2	0.6421	0.8465	0.7308	0.8994	0.9652
	R	0.8013	0.9200	0.8549	0.9484	0.9824
AClogP	*a*	0.3636	0.8845	0.5027	0.4118	0.7107
	b	1.3327	1.9478	1.7437	0.8511	2.1039
	R^2	0.6162	0.8106	0.6814	0.8713	0.9428
	R	0.7850	0.9003	0.8255	0.9334	0.9710
XlogP2	*a*	0.3227	0.7694	0.4439	0.3574	0.6286
	b	1.7411	2.9691	2.3058	1.3280	2.8970
	R^2	0.6282	0.7937	0.6743	0.8492	0.9360
	R	0.7926	0.8909	0.8212	0.9215	0.9675
XLOGP3	*a*	0.3356	0.7975	0.4582	0.3706	0.6535
	b	1.1323	1.5267	1.4794	0.6574	1.7108
	R^2	0.6246	0.7840	0.6600	0.8396	0.9293
	R	0.7903	0.8854	0.8124	0.9163	0.9640
$LogP_{ACD}$	*a*	0.3350	0.8365	0.4880	0.3830	0.6814
	b	1.7718	2.9812	2.2952	1.3427	2.8968
	R^2	0.5706	0.7911	0.6717	0.8221	0.9064
	R	0.7554	0.8894	0.8196	0.9067	0.9521

Pearson correlation coefficient for the relation between the values obtained *in-silico* and experimental value R_M^0 are greater than or equal to 0.7554, which means that the correlation between the results is positively high or positively very high. The highest value was obtained for the modifier 1,4-dioxane in ALOGPs and the lowest value for the acetonitrile modifier in $LogP_{ACD}$ and they are 0.9824 and 0.7554, respectively. The highest values of R coefficient were obtained for the modifier 1,4-dioxane in all programs and fall within the scope 0.9521-0.9824. Equally high values can be observed for propan-2-ol. The lowest values were obtained for acetonitrile and fall within the scope 0.7554-0.8013.

Table S13. Regression coefficients and Pearson correlation coefficient for the relation between the values obtained *in-silico* and experimental value *A* (slope of the straight).

A (experimental) = a · LogP + b						
**Programme**	**Regression coefficients**	**acetonitrile**	**methanol**	**acetone**	**propan-2-ol**	**1,4-dioxane**
ALOGPs	*a*	-0.0009	-0.0088	-0.0048	-0.0388	-0.0084
	b	-0.0246	-0.0207	-0.0249	-0.0154	-0.0327
	R^2	0.0391	0.7725	0.5053	0.7389	0.9427
	R	0.1977	0.8789	0.7108	0.8596	0.9709
AClogP	*a*	-0.0007	-0.0073	-0.0039	-0.0032	-0.0071
	b	-0.0255	-0.0269	-0.0285	-0.0181	-0.0384
	R^2	0.0278	0.7305	0.4568	0.6976	0.9177
	R	0.1667	0.8547	0.6759	0.8352	0.9580
XlogP2	*a*	-0.0006	-0.0063	-0.0034	-0.0028	-0.0063
	b	-0.0261	-0.0353	-0.0328	-0.0219	-0.0462
	R^2	0.0335	0.7106	0.4535	0.6682	0.9112
	R	0.1830	0.8430	0.6734	0.8174	0.9546
XLOGP3	*a*	-0.0007	-0.0065	-0.0035	-0.0028	-0.0065
	b	-0.0250	-0.0236	-0.0265	-0.0168	-0.0345
	R^2	0.0319	0.6997	0.4395	0.6554	0.9040

	R	0.1786	0.8365	0.6629	0.8096	0.9508
$LogP_{ACD}$	*a*	-0.0005	-0.0068	-0.0038	-0.0029	-0.0067
	b	-0.0264	-0.0355	-0.0327	-0.0221	-0.0463
	R^2	0.0183	0.7064	0.4686	0.6331	0.8736
	R	0.1353	0.8405	0.6845	0.7957	0.9361

The Pearson correlation coefficient for the relationship between the values obtained *in-silico* and the experimental value *a* (straight slope) varies according to the organic mobile phase modifier used. Negligible correlation was obtained for acetonitrile (R < 0.3000). Moderate positive correlation is visible for most programs when the organic modifier was acetone (0.5000 < R < 0.7000). Other results in all programs are high positive or very high correlation. The highest values of Pearson R were again obtained for 1,4-dioxane and are in the range 0.9361-0.9709.

3. Correlations between in-silico results

Statistical analysis of all LogP values obtained theoretically for all programs and the Molinspiration Internet database was also carried out. All results were compared with each other and R^2 coefficients were calculated. The results are presented in Table S14.

Table S14. Values of R^2 obtained by comparing in-silico results.

		Programme					
		**miLogP**	**ALOGPs**	**AClogP**	**XlogP2**	**XLOGP3**	**$LogP_{ACD}$**
**Programme**	**miLogP**	1	–	–	–	–	–
	**ALOGPs**	0.9911	1	–	–	–	–
	**AClogP**	0.9997	0.9924	1	–	–	–
	**XlogP2**	0.9928	0.9916	0.9928	1	–	–
	**XLOGP3**	0.9934	0.9889	0.9932	0.9996	1	–
	**$LogP_{ACD}$**	0.9810	0.9748	0.9782	0.9916	0.9918	1

Each LogP value determination program uses different algorithms to calculate this value, however, there is a very high correlation between the results obtained. Correlation coefficients R^2 are greater than or equal to 0.9748, which indicates that each software can be successfully used for preliminary assessment of lipophilicity of the analyzed compounds.

References

1. Mukaka, M.M. A guide to appropriate use of correlation coefficient in medical research. *Malawi Med. J.* **2012**, 24, 69–71.
2. Available online: http://www.vcclab.org/lab/alogps/
3. Available online: https://www.organic-chemistry.org/prog/peo/
4. Available online: https://www.ics.uci.edu/~dock/manuals/xlogp2.1/
5. Available online: http://www.sioc-ccbg.ac.cn/skins/ccbgwebsite/software/xlogp3/
6. Available online: https://www.acdlabs.com/
